# Retaining Patients in Care: An Important but Neglected Challenge

**DOI:** 10.9745/GHSP-D-19-00091

**Published:** 2019-03-22

**Authors:** Stephen Hodgins

**Affiliations:** a Editor-in-Chief, Global Health: Science and Practice Journal, and Associate Professor, School of Public Health, University of Alberta, Edmonton, Alberta, Canada.

## Abstract

A hospital-based follow-up program in Uganda helped improve retention of patients in care across a range of health problems. Although the specific approach may not be replicable in other settings, hospitals in Uganda and beyond should consider how they can improve retention of patients requiring long-term care, including for HIV, TB, malnutrition, and noncommunicable diseases.

See related article by Alizadeh.

A challenge all health care systems struggle with is ensuring needed continuity of care. The less robust the system, the more difficult that is. The article by Alizadeh et al., in this issue of GHSP, gives an account of efforts made by a rural district hospital in Kisoro, Uganda, to improve retention of patients across several different services in the hospital including those with HIV, receiving antiretrovirals (ARVs); tuberculosis (TB); severe malnutrition; and other chronic, noncommunicable diseases (NCDs).^1^

The hospital developed a defaulter-tracking program, under which outreach staff would periodically visit clusters of villages once a threshold number of patients lost to follow-up was reached for that cluster, and encourage patients to return for follow-up treatment. The hospital made available a motorcycle for this purpose and offered field staff an incentive for patients successfully found and referred back for care.

## DEFAULTER-TRACKING FOR HIV AND TB

Similar programs have been introduced elsewhere in sub-Saharan Africa. However, as Alizadeh et al. point out, most of these programs have been developed for disease-specific programs, mainly for ARV or TB treatment follow-up.

With funding from sources including the Global Fund to Fight AIDS, Tuberculosis and Malaria and the U.S. President's Emergency Plan for AIDS Relief (PEPFAR) program, HIV treatment has generally had more substantial financial support than other services. Certainly, a strong argument can be made for household and community-level adherence support and other community outreach activities to try to retain ARV patients in treatment. Treatment interruptions lead to shorter life expectancy and increase the need for more expensive second- and third-line treatment regimens.

Similarly, TB programs in Africa have benefited from more external funding than many other programs. There is a compelling public health case for special follow-up measures to maximize TB treatment completion and minimize risk of further transmission and of development of drug resistance. But other conditions—notably NCDs—call for long-term, often lifelong, continued care.

## WHAT DID KISORO DISTRICT HOSPITAL DO?

In the case described in the article by Alizadeh and colleagues, the hospital had access to funds allowing it to develop a more integrated follow-up program, to help improve retention across a range of health problems, including conditions not prioritized by donors. With one full-time coordinator and several part-time outreach workers, in the year for which they provided documentation in their article, they attempted to contact 1,285 patients lost to follow-up, of whom just under two-thirds were located.^1^ Of those found, one-third either had died or were mistakenly included on the list. The outreach service was less effective in tracking down ARV defaulters, finding only half of them.

Of patients found and referred back, over 90% of TB patients returned for follow-up, close to three-quarters of those with NCDs returned, and just over half of the referred ARV patients returned. The authors report this level of performance was considerably better than several years earlier; they attributed this particularly to introduction of the performance-based incentive for the outreach workers.

The annual recurrent cost for this outreach was US$6,600.

## WHAT ABOUT JUST PHONING PATIENTS AND REMINDING THEM OF THEIR APPOINTMENT?

As the authors report, although mobile phone use has increased over the last several years, at the time the follow-up service was introduced phone use was uncommon; therefore, relying on phone contact for follow-up would not have been effective. With mobile phone use now more widespread, in the future a mobile device platform could be used to supplement the face-to-face follow-up model that has been used to date.

Unlike some other programs developed to improve retention and adherence, the service described by Alizadeh and colleagues focused only on face-to-face contact in the community to encourage defaulting patients to return for follow-up; it did not include peer support or other community-based forms of support to address barriers to treatment continuity. Although adding such components could further improve retention in care, clearly this would also cost more money, posing new challenges with regard to sustainability and scalability.

## WHAT IS THE RELEVANCE OF THIS EXPERIENCE ELSEWHERE IN UGANDA, AND BEYOND?

Unlike typical government hospitals in Uganda, Kisoro District Hospital benefited from external financial support, which allowed it to develop this defaulter-tracking program. Generally speaking, government district hospitals have less discretionary money available for such innovations. But one can certainly argue that providing modest additional funds to achieve higher retention in treatment for such cases would be a sound investment, one that could even be cost-saving. As the authors point out, addressing the problem of loss to follow-up using an integrated, multi-service approach is considerably more efficient than implementing separate, disease-specific follow-up programs.

Across different parts of Uganda, conditions vary. So the specific details of an optimal follow-up program will also vary. But giving program attention to improved retention for health issues like ARV treatment, TB, severe malnutrition, and NCDs is not a luxury. Starting patients on treatment and losing them to follow-up is wasteful of resources, results in avoidable bad outcomes for the patients concerned, and—for some conditions (like active TB)—represents a threat to the health of the community.

Giving program attention to achieving improved retention in care for issues like ARV treatment, TB, severe malnutrition, and NCDs is not a luxury.

The specific details of the follow-up scheme used by Kisoro District Hospital may or may not be well-fitted to hospitals in other parts of Uganda and it may or may not be feasible for government to closely replicate such a model. All the same, government, donor partners, and hospital leadership across Uganda and beyond should be asking themselves how they can improve retention of those of their patients requiring long-term follow-up care, not only for the traditionally better-funded HIV and TB programs but also for other conditions needing similar continuity of care.
